# No Evidence That Salt Water Ingestion Kills Adult Mosquitoes (Diptera: Culicidae)

**DOI:** 10.1093/jme/tjaa214

**Published:** 2020-10-20

**Authors:** Donald A Yee, Catherine Dean, Cameron Webb, Jennifer A Henke, Gabriela Perezchica-Harvey, Gregory S White, Ary Faraji, Joshua D Macaluso, Rebecca Christofferson

**Affiliations:** 1 School of Biological, Environmental, and Earth Sciences, University of Southern Mississippi, Hattiesburg, MS; 2 Discipline of Medicine, Westmead Clinical School, Sydney Medical School, University of Sydney, New South Wales, Australia; 3 Medical Entomology, NSW Health Pathology, Westmead Hospital, Westmead, New South Wales, Australia; 4 Coachella Valley Mosquito and Vector Control District, Indio, CA; 5 Salt Lake City Mosquito Abatement District, Salt Lake City, UT; 6 Pathobiological Sciences, School of Veterinary Medicine, Louisiana State University, Baton Rough, LA

**Keywords:** attractive toxic sugar bait, Culicidae, diet, sodium chloride, sucrose

## Abstract

Various products and insecticides are available that purport to reduce wild populations of adult mosquitoes. Recently, several manufacturers and general public comments on the internet have promoted devices that claim that ingestion of salt will significantly reduce populations of wild mosquitoes to near zero; there are no known scientific efficacy data that support these claims. We tested the survival of nine mosquito species of pest and public health importance across four adult diets: Water Only, Sugar Water Only (8.00%), Salt Water Only (1.03%), and Sugar + Salt Water. Species included the following: *Aedes aegypti* (L.), *Aedes albopictus* (Skuse), *Aedes dorsalis* (Meigen), *Aedes notoscriptus* (Skuse), *Aedes vigilax* (Skuse), *Anopheles quadrimaculatus* (Say), *Culex pipiens* (L.), *Culex quinquefasciatus* (Say), and *Culex tarsalis* (Coquillett). Male and female mosquitoes were placed in cages and allowed to feed on liquid diets under controlled environmental conditions for 1 wk. For seven of the nine species, adult survival was significantly higher in the presence (Sugar Water, Sugar + Salt Water) versus the absence (Water Only, Salt Only) of sugar, with no indication that salt had any effect on survival. *Anopheles quadrimaculatus* showed intermediate survival in Sugar + Salt to either Sugar Only or no sugar diets, whereas *Aedes dorsalis* showed low survival in Salt Only versus other diets. Based on our data and coupled with the fact that mosquitoes have physiological and behavioral adaptations that allow them to avoid or process excess salt (as found in blood meals), we conclude that there is no scientific foundation for salt-based control methods of mosquitoes.

The pest and public health risks associated with mosquitoes are significant and are often perceived to be greater where individuals are exposed to mosquitoes in and around residential properties (Halasea et al. 2014). As a result, there continues to be demand for commercial products designed for mosquito control around the home. However, the effective control of mosquitoes has had mixed success. The complex biology and ecology of mosquito species presents challenges in finding an effective and sustainable broad ranging control strategy and the realm of mosquito control has remained relatively static over the past few decades (Faraji and Unlu 2016). Although new technologies and approaches have yielded some success (e.g., attractive toxic sugar baits (e.g., Fiorenzano et al. 2017) and insecticide-treated bed nets (Nahlen et al. 2003), there has been no magic bullet to rid humanity of pestiferous mosquitoes. Over the past few years, there has been growing public interest in novel approaches that purport to help reduce mosquito populations for individual homeowners. One such approach relies on the use of common table salt (sodium chloride) added to a sugar bait to kill adult mosquitoes.

There is a clear demand among the public for affordable and effective mosquito control, and many do-it-yourself approaches have proven popular on internet and social media sites (e.g., YouTube and Facebook). Many of the more widely shared approaches include mixing of various household products alleged to have value as a mosquito attractant, repellent, or control agent. Several devices make claims that salt-based solutions are active killing agents, with some videos describing this approach having millions of views on social media. Within the past few years, several companies have begun to produce devices in the United States that make the claim that salt feeding by adults will reduce mosquito numbers in the wild. These devices include the Spartan Mosquito Eradicator (ACT2 Inc., Hattiesburg, MS), the Mosquito XT (King Marketing, Paragould, AK), the Skeeter Eater (Copia Products, Memphis, TN), Mosquito Dynamite (Vic West Brands, Austin, Texas), and Donaldson Farms – Mosquito Eliminator (Chattanooga, TN). These devices generally contain some combination of dried salt, sugar, and yeast, which is mixed with warm water by the purchaser and then placed outdoors to either attract mosquitoes who then drink the fluid and are claimed to die from the salt, or who are repelled by the action of other additives like various essential oils. There are no data that have tested the effectiveness of salt as a substance to kill mosquitoes.

There are several reasons why salt may be an effective path for mosquito control. First, adult mosquito nutrition is based on the feeding of plant-derived sugars, which also contain a variety of other substances, including proteins, vitamins, amino acids, and salts (reviewed in Peach and Gries 2019). Thus, salts are an essential component of the adult mosquito diet, however one could hypothesize that at high enough concentrations salt could be lethal, although there are little to no data on the effects of such high concentrations on adult survival. A lack of evidence may simply stem from an avoidance by researchers to investigate what is for many a forgone conclusion (i.e., there is no reason to assume that mosquito adults actively drink salt water in nature). Second, eggs of some species often fail to hatch in water with salt concentrations > 1.0% (e.g., Macfie 1922, Wigglesworth 1933) although *Aedes aegypti* (L.) and *Aedes albopictus* (Skuse) show egg hatchings even at 2.0% salt concentrations (sea water is ~3.5% salt; Yee et al. 2013). Third, although salt can be lethal to larvae of nonsalt adapted species (e.g., Yee et al. 2013), larvae of some mosquitoes, such as *Aedes sollicitans* (Walker) and *Aedes taeniorhynchus* (Wiedemann), are known to have a tolerance to salt (Albers and Bradley 2011). Finally, adult females may avoid laying eggs in water with high salt concentrations (e.g., Woodhill 1941, Foley and Bryan 1999). This avoidance to certain salt concentrations is perhaps a way for females to avoid any lethal effects on their offspring. However, despite evidence to suggest that females may be able to detect high concentrations of salt and that salt can be detrimental to larvae and eggs, there remain little data that directly tests the effect of salt ingestion on adult survival. Finally, there is also little information available on the likely ingestion of salt by adult mosquitoes, or other substances in natural sources of sugar that may have adverse effects on adult survival.

We tested the effect of salt on survival in nine species of adult mosquitoes, all having some relevance to human disease and quality of life. Based on the established knowledge about the physiological responses to salt feeding (e.g., Salama 1966, Sheplay and Bradley 1982, Ignell et al. 2010), we hypothesized that low concentrations of salt would not affect adult survival, and we predicted that the addition of salt to a standard sugar diet would not prove to be an effective control mechanism for adult mosquitoes. Given the rise of manufactured products that claim to control mosquitoes via salt feeding, we replicated a set of standard methods across five different research laboratories to test the different species or species complexes of medically important and pestiferous species likely to be encountered by residents around the world.

## Materials and Methods

This study represents the combined contributions of five laboratories who tested species available in their area, and although the methods for the experiments were fundamentally the same, there were slight differences that we highlight by laboratory location (i.e., Australia = AU, USA laboratories are California = CA, Louisiana = LA, Mississippi = MS, and Utah = UT). The nine species included the following: *Aedes aegypti* (L.) (AU, LA), *Ae. albopictus* (Skuse) (MS), *Ae. notoscriptus* (Skuse) (AU), *Ae. dorsalis* (Meigen) (UT), *Ae. vigilax* (Skuse) (AU), *Anopheles quadrimaculatus* (Say) (MS), *Culex pipiens* (L.) (UT), *Cx. quinquefasciatus* (Say) (CA), and *Cx. tarsalis* (Coquillett) (CA). All species are important known vectors of pathogens or nuisance-biting pest species and often are a main focus of vector control and suppression.

### Mosquito Colonies

Adult mosquitoes used in all experiments (except *Aedes dorsalis*) were from colonies maintained in each laboratory using similar rearing and husbandry protocols (Table 1). Unless otherwise noted, the environmental conditions for larvae were the same for all feeding trials (detailed below). *Aedes aegypti* (LA) were reared under 28°C. *Aedes dorsalis* were wild caught females that were trapped in field cages using CO_2_ as bait and supplied overnight with water but no sugar until the next day when trials began. *Aedes vigilax* were reared in diluted seawater with deionized water to a salinity of ~16 ppK. *Anopheles quadrimaculatus* were purchased as eggs from Benzon Research, Inc. (Cumberland County, PA).

**Table 1.** Details of mosquito species used to test the effect of salt on survival

Species, location	Generation	Origin	Larval diet	Blood source
*Aedes aegypti*, AU	Unknown (in colony since 1980s)	AU	Ground fish flakes and brewer’s yeast	*Rattus norvegicus*, Western Sydney Local Health District and University of Sydney animal ethics approval number 8001/04–10
*Ae. aegypti*, LA	Unknown	Rockefeller strain	Ground fish food	Hemotek membrane system with bovine blood
*Aedes albopictus*, MS	F_1_	Hattiesburg, MS	Puppy chow (Purina, Inc.)	Japanese quail, *Coturnix japonica*, IACUC #11092207
*Aedes dorsalis*, UT	Wild caught adults	Salt Lake City, UT	None	None
*Aedes notoscriptus*, AU	Unknown (in colony since 2020)	Sydney, AU	Equal parts brewer’s yeast and fish flakes	*Rattus norvegicus* under Western Sydney Local Health District and University of Sydney animal ethics approval number 8001/04–10
*Aedes vigilax*, AU	Unknown (in colony since 1986)	Townsville, AU	Equal parts brewer’s yeast and fish flakes	*Rattus norvegicus* under Western Sydney Local Health District and University of Sydney animal ethics approval number 8001/04–10
*Anopheles quadrimaculatus*, MS	Unknown (in colony since 2011)	Gainesville, FL	Mixture of yeast and lactalbumin	None
*Culex pipiens*, UT	Unknown (in colony since 2016)	Salt Lake City, UT	Ground rabbit pellets	Hemotek membrane system with bovine blood
*Culex quinquefasciatus*, CA	Unknown (in colony since 1950s)	Merced, CA	Fish flakes, liver powder, yeast, and ground alfalfa pellets	Ring-neck doves, *Streptopelia capicol*
*Culex tarsalis*, CA	Unknown (in colony since 1950s)	Bakersfield, CA	Fish flakes, liver powder, yeast, and ground alfalfa pellets	Ring-neck doves, *Streptopelia capicol*

For each species, we list the laboratory location (Australia = AU, United States includes California = CA, Louisiana = LA, Mississippi = MS, and Utah = UT) where the trials were conducted, the generation of the mosquitoes, their origin, the diet for larval rearing, and the blood source for adults when used to produced eggs.

### Feeding Trials

All locations used similar environmental conditions for larval rearing and feeding trials (unless noted), with feeding trials conducted either in walk-in or separate smaller environmental chambers kept at 27°C (28°C in the case of LA) on a 12:12 light:dark cycle (UT and MS used a 1 h transition from light to dark and dark to light to reflect natural conditions). Humidity was maintained between 50 and 75%. Cages were approximately 30 × 30 × 30 cm and were of either a metal or plastic frame with mesh covering all sides, with the exception of LA who used one quart paper cylindrical containers (Stanpac, Inc. Ontario, Canada). Into each cage, we added 20 adult mosquitoes (10 males and 10 females) each 1–7 d old. Based on the availability of adults, UT used 17–23 total adults for trials, although an approximately equal sex ratio was still maintained and for *Ae. dorsalis* only females were used. In the time between eclosion and the start of the trials (≤1 wk), adults were fed on a 10% sucrose solution ad libitum under similar conditions as the feeding trials. Female *Aedes dorsalis* were subjected to feeding trials the day after they were collected from the wild.

For the feeding trials, we used four no-choice diets (i.e., adults in each cage only had access to one of the four diets): Water Only (negative control), Salt Water Only (1.03% sodium chloride in water, hereafter Salt Only), Sugar Water Only (8% sucrose in water, hereafter Sugar Only), and Sugar + Salt Water (1.03% sodium chloride and 8% sucrose in water, hereafter Sugar + Salt). Percentages used were based on the product description from the most widely available commercial product (Spartan Mosquito Eradicator) but are similar to other available products. These percentages reflect those found after filling the container with fluid per the manufacturer’s directions and not the percentages of dried product. Mosquitoes are commonly fed a 10% sucrose solution in colonies under laboratory conditions. We replicated each diet three times for each species. Liquid for each diet was added fresh on day 1 of the experiment and replaced on day 4. Diets were added to vials with an exposed cotton wick. The trials ran for 7 d and on each day, we recorded the number of dead mosquitoes of each sex. Within each laboratory, all diets were run concurrently for each species.

### Statistical Analysis

Survival analyses were conducted for each species, separately, using PROC PHREG in SAS (2004). Individuals alive regardless of sex at the end of the experiment yielded censored observations, which are accounted for by the analysis. The overall model considered differences among all diets, but was not capable of determining where specific differences existed. To determine differences between diets (e.g., Salt Only vs Sugar Only), we conducted pair-wise comparisons and adjusted the final *P* value to account for multiple comparisons (*P* = 0.05/6 contrasts = 0.008). We did not analyze sex as a separate factor given that none of the claims made by any of the manufactures of the devices mentioned above suggest sex-specific results of salt feeding, nor did we expect that males and females would differ in their tolerance to ingestion of salt water.

## Results

**Fig. 1.**
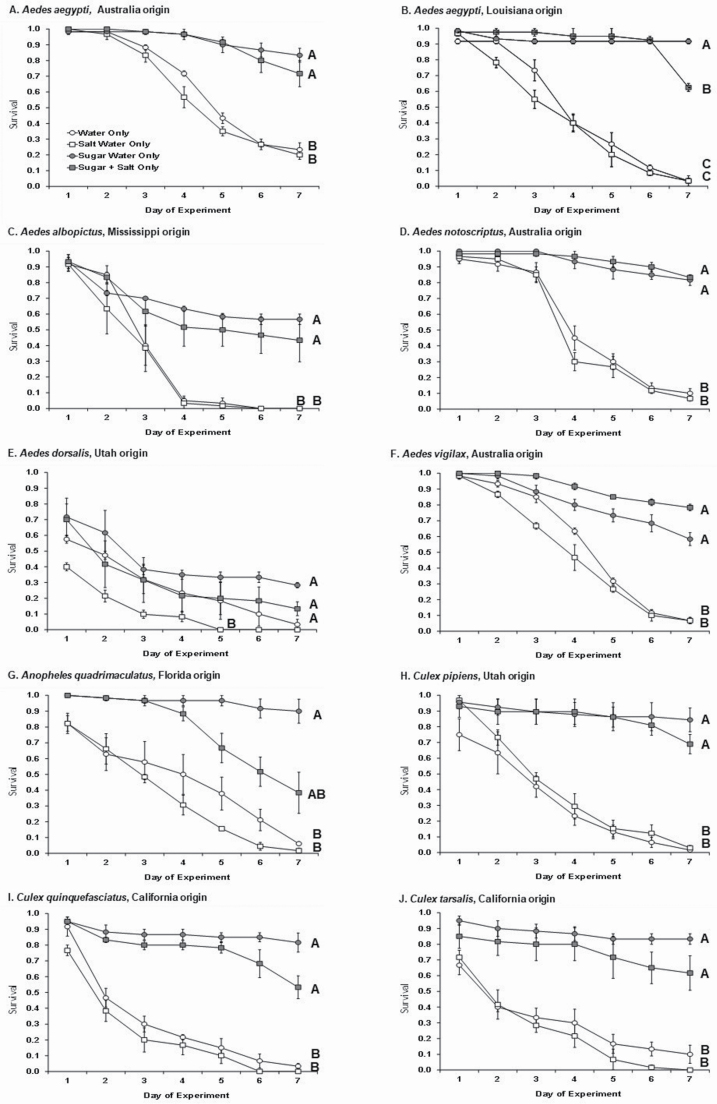
Survivorship curves (mean ± 1 SE) across 7 d for mosquito species across different diets (open circle = Water Only, open square = Salt Water Only, gray circle = Sugar Water Only, gray square = Sugar + Salt Water). For each species (A–J), the origin of that population is listed as in Table 1. Curves that share a letter are not significantly different.

We found significant effects of diet on survival after 7 d of feeding for all species (Table 2). Based on pair-wise comparisons between diets, we generally found significant differences between two sets of diets: those with sugar (Sugar Only, Sugar + Salt), with 7 d survival ranging from 60 to 90%, and those without sugar (Water Only, Salt Only), with 7 d survival ranging from 0 to 20% (Fig. 1A–D, F, H–J). The two exceptions to this were for *An. quadrimaculatus*, which showed intermediate survival in Salt + Sugar compared to either Sugar Only (highest survival), or to Salt Only or Water Only (lowest survival) (Fig. 1G), and for *Ae. dorsalis* wild females which had the lowest survival in Salt Only compared to all other diets (Fig. 1E). However, the addition of salt to sugar never led to any species of mosquito to die at a faster rate compared to sugar alone, with the minor exception of a 1-d difference in the LA *Ae. aegypti* where survival in Salt + Sugar was lower than Sugar Only on day 7 (Fig. 1B). Among the genera, *Aedes* divergence in adult survival in diets with sugar compared to those without sugar often occurred between day 3 and 4 of the experiment (Fig. 1A–D, F), whereas for *Culex*, differences in survival between sugar and no sugar diets were apparent almost from the start of the experiment (Fig. 1H–J).

**Table 2.** Results of survival analysis for mosquito species reared across different diet environments

Species	Location	χ ^2^, df	*P* value
*Aedes aegypti*	AU	60.27, 3	<0.001
*Aedes aegypti*	LA	89.91, 3	<0.001
*Aedes albopictus*	MS	46.74, 3	<0.001
*Aedes dorsalis*	UT	22.48, 3	<0.001
*Aedes notoscriptus*	AU	91.06, 3	<0.001
*Aedes vigilax*	AU	54.11, 3	<0.001
*Anopheles quadrimaculatus*	MS	59.97, 3	<0.001
*Culex pipiens*	UT	102.62, 3	<0.001
*Culex quinquefasciatus*	CA	107.28, 3	<0.001
*Culex tarsalis*	CA	85.11, 3	<0.001

Laboratories where each species were tested are included (Australia = AU, United Sates includes California = CA, Louisiana = LA, Mississippi = MS, and Utah = UT).

## Discussion

Our results from testing the effect of salt on the survival of nine mosquito species of public health importance were clear: There was no evidence from these trials that ingestion of salt had an added lethal effect on adult mosquitoes. Thus, our data support the hypothesis that low concentrations of salt would not affect adult mosquito survival. In seven out of nine of our species, we found that mosquitoes that ingested a diet with salt and sugar survived at rates equal to those fed a standard diet of sugar alone. In the cases where this did not occur, the addition of salt caused increased mortality, but in *An. quadrimaculatus*, 40% of adults were still alive at the end of the experiment. For *Aedes aegypti* from the LA colony, the difference in Salt Only and Salt + Sugar diets was only apparent on the last day of the trial. For all but one species, a diet with only saltwater did not lead to higher mortality rates than water alone, and thus this strongly suggests that salt by itself is not a detrimental substance for the mosquito digestive system. *Aedes dorsalis* did show that a salt only diet led to higher mortality compared to all other diets (including Salt + Sugar); however, on the very first day of the experiment, this diet had the lowest overall survival across all species tested, and we note that survival overall was low across all diets (Fig. 1E). Unlike the other species that were reared from larvae in the laboratory, all *Ae. dorsalis* females were wild caught and of unknown age and of unknown sugar feeding status, and thus the higher overall mortality on day 1 could reflect general attrition due to acclamation to laboratory conditions. However, we still did not find evidence that a salt and sugar diet compared to sugar only was detrimental to this species.

It is important to note that mosquitoes often are exposed to salt in nature as part of their normal diet. Adult mosquitoes often ingest salts as a component of plant-derived sugars (reviewed in Peach and Gries 2019) as well as blood (Clements 2000). Human blood is 0.9% salt and is commonly ingested by females of many species of mosquitoes, including those tested here, to complete egg production. However, mosquitoes have physiological mechanisms that allow them to deal with excess salt from blood meals. Specifically, salts like Na+, K+, and Cl−, are first absorbed across the stomach and are then rapidly eliminated by Malpighian tubules with coordinated actions of the hindgut (Bradley 1987). In addition, after an adult female ingests a bloodmeal, they produce copious urine, which is more sodium rich than that produced at other times. This diuresis rids females of 40% of the water, Na+, and Cl− in the ingested bloodmeal, and 20% of the ingested weight (Sheplay and Bradley 1982). Thus, salt ingestion by adults, perhaps even in levels exceeding those found in human blood, are unlikely to lead to increased mortality given that any detrimental effects are countered with physiological adaptations that adults already possess. We based our salt concentration (1.03%) on product values listed on the most widely available commercial product (Spartan Mosquito Eradicator), and note that this salt concentration is approximately the same that is found in human blood (0.9%). Thus, we can see no way that such a concentration would kill adult mosquitoes given that countless adult female mosquitoes have successfully taken a human bloodmeal and survived to produce prodigious progeny. Indeed, salt water generally had the same effect on adult survival as water alone, providing further evidence that approximately 1% salt is not an effective agent of mosquito mortality.

Besides the ability to deal with excess salt ingested during feeding, female mosquitoes also have been shown to simply avoid high salt fluids. Salt detection itself is crucial for maintaining both the ionic drive across the gut and maintaining the homeostatic environment of the hemolymph (Salama 1966). Adult mosquitoes can detect salt in water using tarsal segments, which is likely how they evaluate ingesting a nutrient source they touch (Christophers 1960, Salama 1966). Ignell et al. (2010) showed that *Ae. aegypti* rejected diets containing high salt. Specifically, when offered a choice between varying concentrations of sucrose and sucrose and salt, fewer mosquitoes partook of the sucrose with added salt. The response appeared to be bimodal based on salt concentration, with more feeding on sucrose only in concentrations with either higher or lower than 1 mM salt (Ingnell et al. 2010). Gonzales and Hansen (2016) demonstrated that sucrose meals including either NaCl or CaCl_2_ had higher median rejection thresholds by adults compared to other salts and other chemicals (e.g., HCl). This suggests that mosquitoes, in this case *Ae. aegypti*, reject sucrose solutions that contain high concentrations of salts relative to natural sugar meals. However, there are also data to suggest that salts may be an important stimulatory factor for adult feeding. For *Cx. pipiens*, NaCl at 150 mM acted as a phagostimulant (Hosoi 1959) and meals containing sodium chloride and sodium bicarbonate offered to *Anopheles stephensi* (Liston), *An. freeborni* (Aitken), and *An. dirus* (Petron and Harrison) all elicited greater feeding, indicating that these chemicals were phagostimulatory (Galun et al. 1985); none of these studies reported that higher ingestion of salt led to higher mortality. Even if salt can act to increase feeding, there is no support from our results that it causes increased mortality for the medically important species tested.

In addition to direct feeding on salt, there has been research to investigate how salt may affect other mosquito life history stages and activities, specifically in terms of egg hatching (e.g., Osborn et al. 2006, Yee et al. 2013), larval survival and growth (Wigglesworth 1933, Bañez 1963, Lee 1973, Ramasamy et al. 2011, Albers and Bradley 2011), and oviposition behavior (Woodhill 1941, Wallis 1954, Foley and Bryan 1999, Navarro et al. 2003). Although many species of both saline tolerant and freshwater species have been evaluated, none of these studies appear to suggest that salt is lethal at low concentrations (e.g., <1.00%) to eggs or larvae. Furthermore, where salt has been shown to affect some aspect of life history (eggs, Yee et al. 2013), it also may modify behavior (e.g., oviposition) away from locations with high salt (Woodhill 1941, Navarro et al. 2003).

Although devices currently marketed on social media and by some manufacturers would appear to be, “too good to be true,” consumers have already spent millions of dollars purchasing them, perhaps at the expense of known effective approaches to killing adult mosquitoes. Recent work by Aryaprema et al. (2020) found no evidence that the Spartan Mosquito Eradicator reduces populations of *Aedes albopictus* under controlled laboratory and field conditions. These authors did not test the potential killing action of salt, but focused on the efficacy of the entire product, which also makes other claims (e.g., mosquitoes are attracted CO_2_ produced via fermentation by yeast). Our data specifically addressing the effect of salt ingestion appear to support the conclusion of Aryaprema et al. (2020) that the Spartan Mosquito Eradicator in its present formulation does not reduce mosquito populations. In particular, we find no evidence that salt ingestion in adult mosquitoes is an effective control approach.

As in many instances state and federal laws do not require efficacy data to support claims made by these devices, it is important to evaluate individual claims to better inform the public and ensure that limited public health dollars are not needlessly wasted on approaches that do not effectively control mosquitoes (Revay et al. 2013). We would also caution that relying on an approach that has no scientific basis may result in a false sense of security for homeowners, which may be dangerous in areas where mosquitoes could potentially be transmitting pathogens.

Based on the response of nine medically important species of mosquitoes to different diets, and the substantial literature on the physiological and behavioral ways that mosquitoes deal with salt in nature, we can conclude that there is no scientific or experimental evidence to support the claims that salt-based approaches are effective for mosquito control as currently formulated. As adult mosquitoes do not appear to suffer mortality from ingesting low doses of salt in their diet, and higher concentrations of salt can be detected and avoided by adults, we conclude that salt is ineffective for the control of mosquito populations by individual consumers, regulatory agencies, or mosquito control districts.
